# The Effect of Dexamethasone on Postoperative Pain Management in Patients Undergoing Total Knee Arthroplasty: A Randomized Controlled Trial

**DOI:** 10.7759/cureus.37052

**Published:** 2023-04-03

**Authors:** Junaid Khan, Raja Adnan Ashraf, Hafiz Muhammad Bilal Shabbir, Ali Haider, Sajeel Saeed, Abdul Rauf Khalid, Haroon Shabbir, Syed Naqash Haider Kazmi, Khawar Abbas, Jawad Basit

**Affiliations:** 1 Orthopedic Surgery, Benazir Bhutto Hospital, Rawalpindi, PAK; 2 Orthopedics and Traumatology, Islamic International Medical College, Rawalpindi, PAK; 3 Internal Medicine, Benazir Bhutto Hospital, Rawalpindi, PAK; 4 Internal Medicine, Hamdard College of Medicine and Dentistry, Karachi, PAK; 5 Surgery, Holy Family Hospital, Rawalpindi, PAK; 6 Orthopedic Surgery, Holy Family Hospital, Rawalpindi, PAK; 7 Pediatric Surgery, Benazir Bhutto Hospital, Rawalpindi, PAK; 8 Cardiology, Holy Family Hospital, Rawalpindi, PAK

**Keywords:** dexamethasone, total knee arthroplasty (tka), total knee replacement (tkr), pain management, visual analog pain scale

## Abstract

Objective

The objective of this study was to evaluate the effectiveness of dexamethasone in treating postoperative pain in patients undergoing total knee arthroplasty (TKA).

Methodology

This randomized controlled trial (RCT) was completed over the course of two years (September 7, 2015, to September 6, 2017). In the course of their treatment for osteoarthritis of the knee, all of the patients who had primary unilateral total knee replacement (TKR) participated in the research. Under spinal anesthesia, each patient had medial para-patellar approach medial orthopedic surgery. Patients were assigned to group A or group B based on a random selection. Each of the groups consisted of 79 individuals. Group A was given dexamethasone through intravenous administration at a dose of 0.1 mg/kg before the operation. During the subsequent period of 24 hours, no more treatment was administered (control group). On a predesigned questionnaire, postoperative pain was measured using the visual analog scale (VAS) for pain. Functional results, duration of hospital stay, and complications were all recorded on the questionnaire (VAS). Analysis of data was carried out using the Statistical Package for the Social Sciences (SPSS) version 23 (IBM SPSS Statistics, Armonk, NY, USA).

Results

There were 158 patients in total in the study, out of which 98 were females and 60 were males in the group. The patients’ average body mass index (BMI) was 26.94 ± 3.14 kg/m^2^. Patients in group A had lower postoperative analgesic and antiemetic needs and higher VAS scores and spent less time in the hospital than patients in group B. There were no postoperative problems in either group.

Conclusion

In patients undergoing TKA, the use of dexamethasone during and after surgery decreases pain, the need for analgesics, and the duration of hospital stay.

## Introduction

Total knee arthroplasty (TKA) has received widespread acceptance as the preferred method of treating advanced knee arthritic disease [1]. Osteoarthritis of the knee is a degenerative condition that affects the articular cartilage and is linked to lifestyle factors, gender, age, and obesity. End-stage osteoarthritis of the knee may be surgically treated with osteotomy, unicompartmental knee arthroplasty (UKA), and total knee arthroplasty (TKA) [2]. When all other options for treating the arthritic condition have been explored, TKA is an elective operation. Although it is often regarded as a safe and economical operation, sepsis, extra-articular infections, and vascular problems make it contraindicated. There are many surgical methods for TKA, with the medial para-patellar route being the most common [1]. Being one of the most popular orthopedic procedures, the clinical outcomes of TKA based on patient satisfaction and quality of life improvement are crucial to evaluate. Usually, moderate to severe postoperative discomfort follows TKA. Multimodal analgesic regimens, including nonsteroidal anti-inflammatory drugs (NSAIDs), opioids, local anesthetics, physical therapy, regional anesthesia including continuous nerve catheters for the immediate postoperative period, and cognitive behavioral therapy, have been employed for the management of postoperative pain in total knee arthroplasty [3]. The care for this postoperative pain, however, is not standardized owing to the diversity of patient characteristics.

In recent years, there have been more and more discussions on how to effectively manage postoperative pain after TKA, with the goals of enhancing clinical results, regaining the knee’s functional mobility, and rehabilitating patients who have had this treatment. In our randomized controlled trial (RCT), we want to know how dexamethasone, a glucocorticoid drug, affects postoperative pain after TKA. Dexamethasone is a long-lasting glucocorticoid anti-inflammatory drug that works through phospholipase inhibition. The quantity of pain stimulants produced by the lipoxygenase and cyclooxygenase pathways is thus reduced by this inhibition. Its anti-inflammatory effects are mainly noticeable 24-48 hours after treatment, and its half-life is 36-55 hours [4]. Although the recommended dosage of dexamethasone has not yet been established, several studies have shown substantial improvements in patient clinical outcomes and lower pain ratings as a result of the pre-, peri-, and postoperative administration of dexamethasone [4-6]. Dexamethasone therapy also reduces the frequency of postoperative nausea and vomiting (PONV), according to studies [7]. Dexamethasone also lowers the immune system, which increases the risk of postoperative infections in the patient [8].

Following TKA, patient satisfaction is correlated with preoperative expectations, mental health, functional abilities, and postoperative pain [9]. Opioids have traditionally been used to treat this pain, but extended use of these drugs may result in reliance and addiction [10]. The use of dexamethasone, which has demonstrated lower pain ratings in the early postoperative days, may help prevent the development of chronic pain after TKA [4-8]. This RCT would be a valuable addition to the currently available literature on pain management following TKA, by demonstrating the effects of dexamethasone on postoperative pain, duration of hospital stays, opioid consumption, and use of analgesics.

## Materials and methods

Study design and study setting

The aim of this single-center, randomized, double-blind, placebo-controlled experiment was to assess the effects of dexamethasone on postoperative pain in patients following primary total knee arthroplasty. This randomized controlled trial (RCT) was conducted in the Department of Orthopedics at Benazir Bhutto Hospital in Rawalpindi, Pakistan, from September 7, 2015, to September 6, 2017.

Ethical approval

The criteria and requirements outlined in the Standards for Reporting Qualitative Research were taken into consideration while carrying out this research [11]. Before the research could be carried out and data could be collected, the Ethical Review Board of Rawalpindi Medical University provided its clearance on an ethical level (approval number: S-32-45-22). The research was conducted in compliance with the 1983 revision of the 1975 Helsinki Declaration. In all instances, written informed permission was acquired from patients or their families.

Intervention

**Figure 1:**
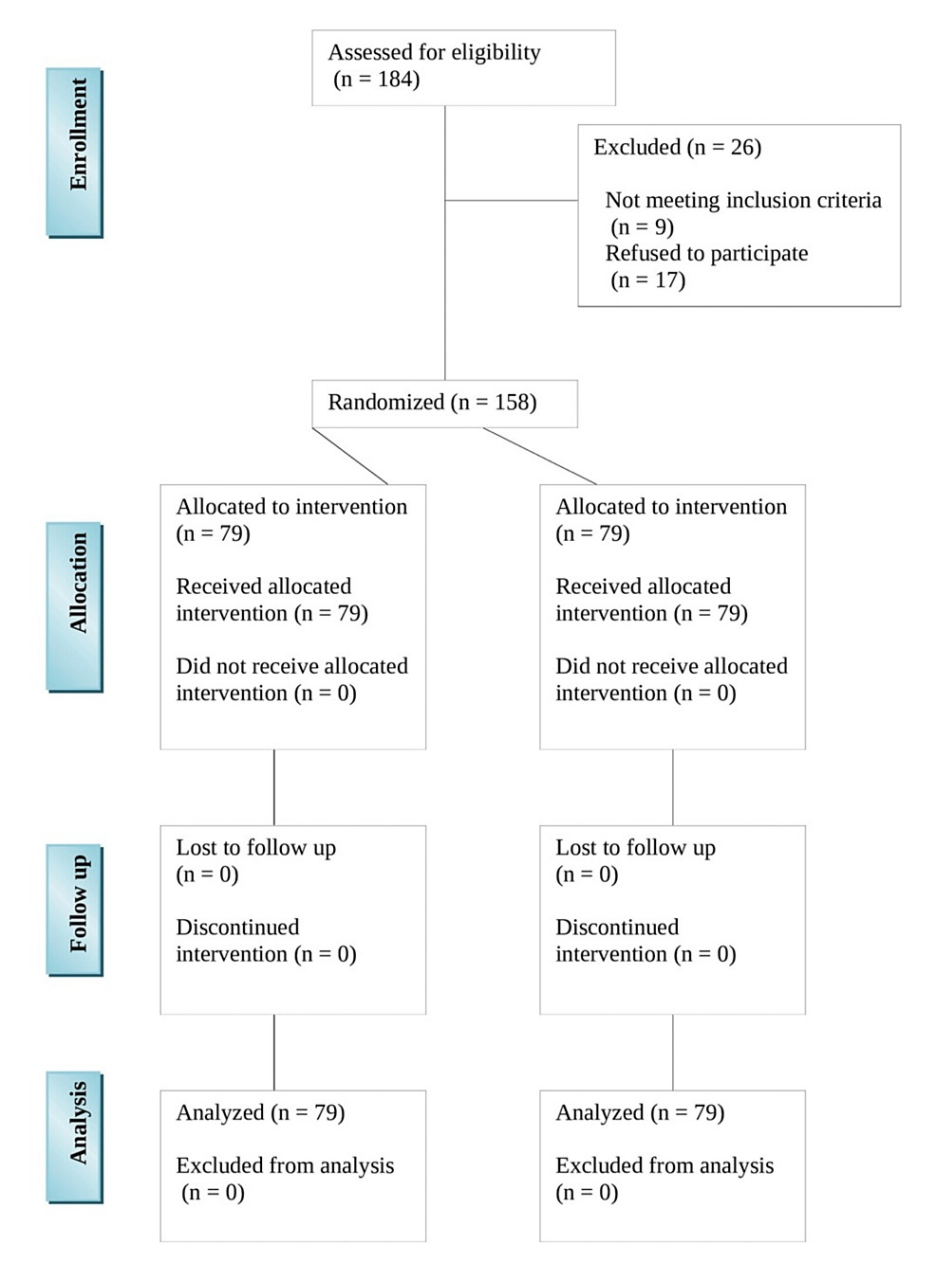
CONSORT diagram showing the flow of participants through each stage of the randomized trial

After the recruitment of the participants, the nature of the research, as well as its goals, was explained to each participant, and their informed consent was obtained appropriately. It was required that the consent form be filled out before proceeding with the remainder of the answer form. The consent form was included as part of the response questionnaire. An external trial partner used a computer-generated randomized sequence (randomisation.com) with different block sizes (two, four, or six) that were undisclosed to the investigators, as well as site stratification, to randomize the groups in a 1:1 design. The patients were subsequently assigned to one of two groups: group A received dexamethasone, while group B served as control. There were 79 patients in each group. Those in group A were given dexamethasone intravenously at 0.1 mg/kg 15 minutes before the operation, as well as another dosage 24 hours after the procedure, while participants in group B did not receive any dexamethasone at all. The intervention was concealed from participants, personnel, investigators, surgeons, outcome assessors, statisticians, and those drawing conclusions (Figure 1).

Inclusion and exclusion criteria

Patients with hepatic or renal failure, poor glycemic control (hemoglobin A1c (HbA1c) > 7.6), corticosteroid or immunosuppressive medication use in the past six months, or a known mental disorder were excluded from the trial. The research comprised patients receiving primary unilateral total knee replacement (TKR) for knee osteoarthritis.

Study questionnaire and data collection technique

Consideration was given to postoperative pain assessed using a visual analog scale (VAS) for pain, functional outcome, length of hospital stays, and complications documented on a predesigned questionnaire.

Statistical analysis

The Statistical Package for the Social Sciences (SPSS) version 23 (IBM SPSS Statistics, Armonk, NY, USA) was used throughout the data analysis process. To establish the mean age, gender distribution, mean body mass index (BMI), and distribution of patients according to the American Society of Anesthesiologists (ASA) physical status categorization, descriptive statistics were employed. Independent t-test and chi-square test were used to statistically compare the difference between demographic variables. To ascertain whether or not the use of dexamethasone for pain management is significant, a chi-square test was carried out. In addition, the same test was carried out to evaluate the importance of using dexamethasone to reduce one’s use of opioids. To be declared statistically significant, the P value has to be lower than 0.05. Graphs were created using Microsoft Excel (Microsoft® Corp., Redmond, WA, USA).

## Results

Demographic characteristics

Out of the 158 participants, half of them (50%) were included in group A (dexamethasone group) and half of them (50%) were included in group B (control group). The average ages of group A and group B patients were 61.12 ± 7.04 and 60.79 ± 6.14, respectively. As for gender, 98 (62.02%) were females and 60 (37.98%) were males. The average BMI of patients was 26.94 ± 3.14 kg/m^2^. According to ASA physical status classification, out of 158 patients, only two patients fell into ASA class I, 158 patients fell into ASA class II, and 38 patients were in ASA class III. No significant difference was found between the demographic variables (Table 1).

**Table 1:** Demographic characteristics of dexamethasone and control group ASA: American Society of Anesthesiologists, BMI: body mass index

Characteristics		Group A (dexamethasone group) (n = 79)	Group B (control group) (n = 79)	P value
Age (years)		61.12 ± 7.4	60.79 ± 6.14	0.77
Gender	Male	29 (36.7%)	31 (39.2%)	0.48
	Female	50 (63.3%)	48 (60.8%)	
BMI (kg/m^2^)		26.94 ± 3.1	27.11 ± 3.3	0.69
ASA classification	I	1 (1.26%)	1 (1.26%)	0.55
	II	58 (73.41%)	60 (75.94%)	
	III	20 (25.31%)	18 (22.78%)	

Comparison of groups A and B according to pain scale (VAS score)

**Figure 2:**
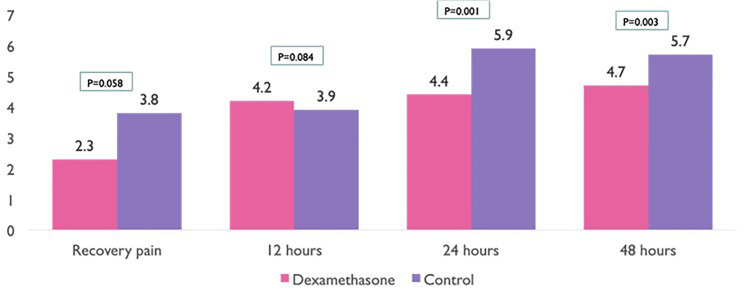
Comparison of groups A and B according to pain scale (VAS score) On the x-axis, recordings of VAS scores at certain times (immediately after surgery, 12 hours, 24 hours, and 48 hours) are given, whereas on the y-axis, VAS score levels are given. VAS: visual analog scale

Patients in group A had better VAS scores (P < 0.05). At three time points, immediately after surgery (recovery time), 24 hours, and 48 hours, a lower VAS score was recorded in group A participants as compared to group B participants, i.e., a considerable difference in pain perception existed between the two groups. This means that a significant statistical correlation is present between pain management and the efficacy of dexamethasone. At 12 hours after surgery, a slightly higher VAS score was recorded among group B participants as compared to group A participants (Figure 2). Also, group A participants had shorter hospital stays (P < 0.05) as compared to group B participants.

Comparison of groups A and B according to opioid consumption

**Figure 3:**
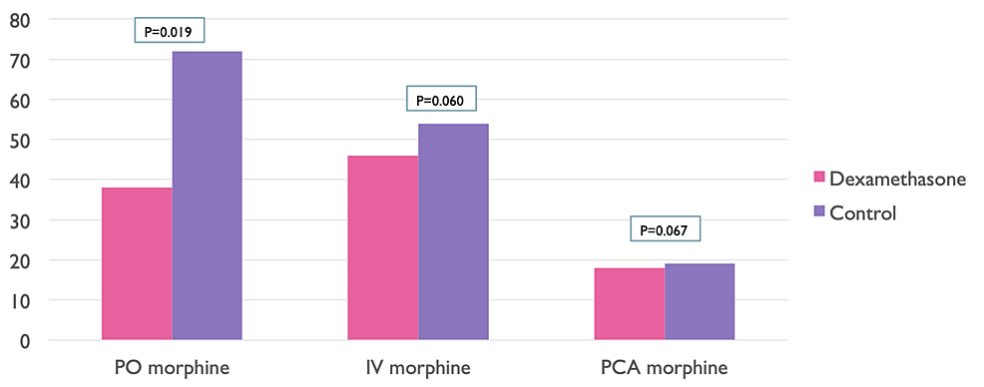
Comparison of groups A and B according to opioid consumption On the x-axis, per oral morphine, intravenous morphine, and patient-controlled analgesia morphine were given, whereas on the y-axis, the quantity of dose was given. PO: per oral, IV: intravenous, PCA: patient-controlled analgesia

Patients in group A required a smaller amount of postoperative analgesics (P < 0.05). Less opioid consumption was observed in group A as compared to group B for oral opioids (per oral morphine) and systemic opioids (intravenous morphine), whereas there was a slight difference in the consumption of patient-controlled analgesia (PCA morphine) among both groups (Figure 3). Also, fewer antiemetics (P < 0.05) were required for group A participants as compared to group B participants.

## Discussion

Quick surgical rehabilitation and successful patient recovery depend on effective pain management. Moreover, postoperative pain can result in delayed discharge, prolonged stiffness, complications of immobility, inability to execute rehabilitation exercises, delayed recovery, poor outcomes, and higher use of healthcare resources.

Our randomized controlled trial (RCT) showed significant improvements in clinical outcomes and patient satisfaction levels following the administration of dexamethasone in patients undergoing total knee arthroplasty (TKA). No postoperative complications following the administration of dexamethasone were observed. This supports and adds to the currently available literature regarding the safety and efficacy of dexamethasone as a viable treatment option for postoperative pain in TKA. Postoperative pain reduction is due to the anti-inflammatory effect of steroids. Our trial showed significant reductions in pain scores in the intervention group, 24 and 48 hours postoperation, associated with the administration of dexamethasone. Due to decreased postoperative pain, patient satisfaction is achieved, and health conditions can be trusted.

For our study, dexamethasone was administered pre- and postoperatively, to achieve the maximum effectiveness of the drug. A study out of the People’s Republic of China has demonstrated that a single preemptive high dose of dexamethasone has shown greater effectiveness as compared to two low doses [12]. Another such study has demonstrated that preemptive low-dose dexamethasone reduced the incidence of postoperative emesis and pain following TKA [7]. However, according to a subgroup analysis of a meta-analysis published in 2022, there were no significant differences between single-dose and repeat-dose groups. This meta-analysis also supported the perioperative use of dexamethasone in patients undergoing TKA [13]. Hence, further studies with larger sample sizes need to be conducted to determine the standard dosage and time of administration of dexamethasone, so it can be added to the multimodal analgesic regimen for the management of postoperative pain following TKA.

Additionally, our research showed that after dexamethasone treatment, opioid usage dramatically reduced. This is in accord with the existing body of research on the subject [4]. Dexamethasone as an adjuvant to other multimodal analgesics following total knee arthroplasty decreased morphine usage, according to an RCT conducted in five Danish hospitals [14]. Dexamethasone usage is linked to a shorter hospital stay, which indicates a better prognosis and increased functional mobility of the knee joint in relation to lower VAS ratings, according to new research from our RCT. It also corroborated recent research on reduced postoperative nausea and vomiting (PONV) after dexamethasone treatment for TKA [7]. Dexamethasone’s anti-inflammatory properties may have contributed to a decrease in nausea and vomiting by reducing the postoperative increase in serum indicators of systemic inflammation. Dexamethasone may help lessen the drowsiness, nausea, and dizziness brought on by opioid usage [14]. Dexamethasone decreased postoperative pain, postoperative nausea and vomiting, and total opioid usage, according to a meta-analysis [15]. Preoperative dexamethasone decreased postoperative pain and opiate use, increased mobility, and enhanced recovery quality after total knee arthroplasty, according to randomized controlled research [16]. Dexamethasone decreased moderately to severe pain in high-pain responders after surgery, according to a different trial [17]. Dexamethasone treatment enhances the postoperative range of motion, according to another meta-analysis, which might be explained by the steroid’s painkilling effects [18]. The long-term administration of glucocorticoids and other steroid medications postoperatively has been associated with an increased risk of hyperglycemia in nondiabetic patients [19] and an increased risk of infection due to poor wound healing [20]. However, more studies need to be done to ascertain whether the long-term adverse effects of dexamethasone administration are comparable in their severity to any possible short-term adverse effects of a single-dose administered peri- or postoperatively [21].

## Conclusions

Being an elective procedure, patient satisfaction following total knee arthroplasty remains an important factor for surgeons to consider when recommending this procedure as a treatment option for knee osteoarthritis. Patient satisfaction depends upon a number of factors, including restoration of the functional motility of the knee and postoperative pain management. According to our study, the use of dexamethasone pre- and postoperatively has shown improved patient pain scores, has reduced the usage of opioids and analgesics, and has resulted in a decreased duration of hospital stay in patients following TKA. However, the plan for pain management following any surgical procedure should be diligently devised by the surgeon, keeping in mind individual patient parameters and disease prognosis. Our clinical trial is an addition to the currently available literature regarding postoperative pain management following TKA.
